# A machine learning framework to predict PPCP removal through various wastewater and water reuse treatment trains†

**DOI:** 10.1039/d4ew00892h

**Published:** 2024-12-19

**Authors:** Joung Min Choi, Vineeth Manthapuri, Ishi Keenum, Connor L. Brown, Kang Xia, Chaoqi Chen, Peter J. Vikesland, Matthew F. Blair, Charles Bott, Amy Pruden, Liqing Zhang

**Affiliations:** a Department of Computer Science, Virginia Tech Blacksburg VA 24061 USA lqzhang@cs.vt.edu; b Department of Civil and Environmental Engineering, Virginia Tech Blacksburg VA 24061 USA apruden@vt.edu; c Civil, Environmental and Geospatial Engineering, Michigan Tech University MI 49931 USA; d Genetics, Bioinformatics, and Computational Biology, Virginia Tech Blacksburg VA 24061 USA; e School of Plant and Environmental Sciences Blacksburg VA 24061 USA; f Hampton Roads Sanitation District Virginia Beach VA 23455 USA

## Abstract

The persistence of pharmaceuticals and personal care products (PPCPs) through wastewater treatment and resulting contamination of aquatic environments and drinking water is a pervasive concern, necessitating means of identifying effective treatment strategies for PPCP removal. In this study, we employed machine learning (ML) models to classify 149 PPCPs based on their chemical properties and predict their removal *via* wastewater and water reuse treatment trains. We evaluated two distinct clustering approaches: C1 (clustering based on the most efficient individual treatment process) and C2 (clustering based on the removal pattern of PPCPs across treatments). For this, we grouped PPCPs based on their relative abundances by comparing peak areas measured *via* non-target profiling using ultra-performance liquid chromatography-tandem mass spectrometry through two field-scale treatment trains. The resulting clusters were then classified using Abraham descriptors and log *K*_ow_ as input to the three ML models: support vector machines (SVM), logistic regression, and random forest (RF). SVM achieved the highest accuracy, 79.1%, in predicting PPCP removal. Notably, a 58–75% overlap was observed between the ML clusters of PPCPs and the Abraham descriptor and log *K*_ow_ clusters of PPCPs, indicating the potential of using Abraham descriptors and log *K*_ow_ to predict the fate of PPCPs through various treatment trains. Given the myriad of PPCPs of concern, this approach can supplement information gathered from experimental testing to help optimize the design of wastewater and water reuse treatment trains for PPCP removal.

## Abstract 2

ML Framework for PPCPs fate in WWTPs.

## Graphical abstract

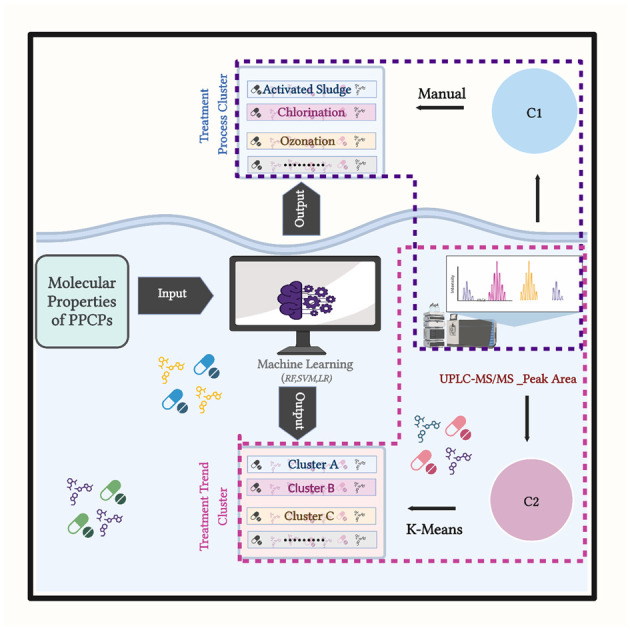

**Water impact**

Here we introduce a machine learning approach to predict the removal of pharmaceuticals and personal care products (PPCPs) during wastewater and water reuse treatment. By reducing the need for costly and labor-intensive analytical testing, this approach supports assessment of treatment efficacy and optimization treatment processes for efficient removal of various PPCPs.

## 1. Introduction

Pharmaceuticals and personal care products (PPCPs), comprising over 4000 diverse natural and synthetic substances, are widely used in medicine, industry, and consumer products.^1–3^ Their extensive use has led to widespread occurrence in water bodies, as conventional wastewater treatment plants are not specifically designed for their removal.^4–6^ This has raised concerns in various water reuse scenarios, including irrigation, groundwater recharge, and indirect potable reuse.^7,8^ The presence of PPCPs in aquatic environments poses ecotoxicological risks, including endocrine disruption and potential human health hazards, even at trace concentrations.^9–12^ These concerns are particularly relevant in water reuse contexts, where there is increased potential for human exposure.^13,14^

The chemical diversity of PPCPs and their typically low concentrations pose significant challenges to identifying effective removal processes.^15–17^ The removal of PPCPs during treatment is influenced by both operational factors and the inherent chemical properties of the compounds, such as molecular weight, hydrophobicity, and charge, which ultimately dictate their fate and removal efficiencies *via* various treatment processes.^18–23^

Abraham descriptors have recently been proposed to categorize the general chemical properties of PPCPs^24–27^ and thus could aid in predicting their removal *via* various treatment processes. These descriptors offer a comprehensive profile of a compound's solvation properties and molecular interactions, which directly relate to whether a PPCP is likely to be removed *via* biological, sorptive, or oxidative processes, *etc.*^28^ By quantifying the molecular interactions that govern sorption processes, Abraham descriptors could provide a systematic framework for understanding and predicting PPCP behavior in various treatment systems.

The advancement of analytical technologies for PPCP detection first brought to light their widespread occurrence in aquatic environments and remain the gold standard for measuring their removal *via* various treatment processes. Over the past two decades, early detection methods like gas chromatography/mass spectrometry (GC/MS) have evolved into non-targeted monitoring approaches such as ultra-performance liquid chromatography tandem mass spectrometry (UPLC-MS/MS).^29–31^ These advanced methods enable the simultaneous analysis of multiple PPCPs with diverse properties, offering a comparative evaluation of removal efficiencies across a wide array of compounds. Conventional monitoring of PPCPs through treatment trains remains essential for regulatory compliance and watershed management. Field measurements provide direct evidence of PPCP occurrence and removal, but are resource-intensive, requiring specialized expertise and costly instrumentation.^32–35^ The increasing complexity of data yielded by advanced analytical technologies like UPLC-MS/MS further complicates interpretation, underscoring the need for approaches to fully leverage this wealth of information. Analytical methods are particularly insufficient for emerging contaminants, where methods are not yet available, or if the aim is to assess a broad spectrum of PPCPs of concern.^36,37^

Recently, machine learning (ML) has shown promise in water and wastewater treatment studies,^38,39^ providing a powerful array of tools for revealing important patterns in complex data sets, including non-linear relationships.^40,41^ However, previous works have employed single ML frameworks, which incurs some drawbacks.^42–44^ Supervised methods alone often struggle with high-dimensional, complex data and may miss important underlying patterns, while unsupervised techniques in isolation lack predictive capabilities. Given the intricate nature of PPCP behavior across various treatment processes, a more comprehensive approach is needed.

In this study, we propose a novel two-step ML approach to characterize and predict PPCP removal in wastewater and water reuse treatment systems. The method begins with unsupervised learning (clustering) to uncover inherent patterns and reduce dimensionality of complex datasets. This crucial step reveals natural groupings of PPCPs without relying on predefined labels. We then leverage these insights in a supervised ML classification phase, establishing quantitative relationships between PPCP properties and removal outcomes. This combined unsupervised-supervised approach is particularly well-suited to complex datasets, revealing otherwise hidden patterns and providing predictive capacity. By addressing the limitations of single-algorithm methods, our framework could provide a more robust and nuanced understanding of PPCP fate during wastewater and reuse treatment. The approach is demonstrated utilizing UPLC-MS/MS data from two full-scale wastewater treatment facilities that are respectively followed by a series of treatments for non-potable and potable reuse.

## 2. Materials and methods

**Fig. 1:**
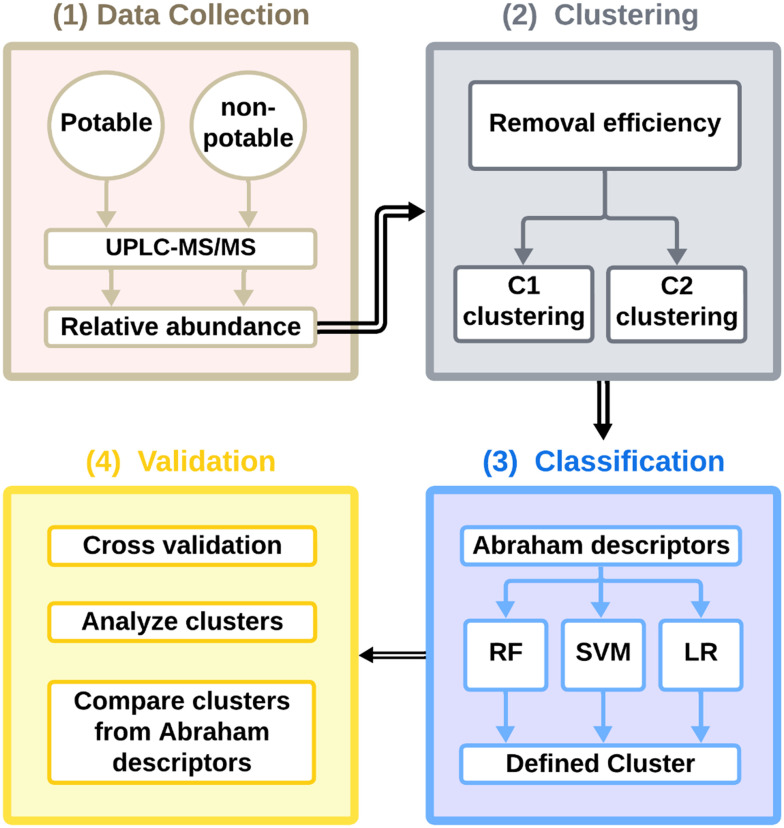
Approach applied to characterize the removal of PPCPs through various wastewater and water reuse treatment processes and to develop the ML framework (RF = random forest, SVM = support vector machines, LR = logistic regression).

This study was conducted in four steps: (1) sampling, analysis, and data collection (2) clustering PPCPs based on their relative abundances through various stages of wastewater and water reuse treatment (3) classification of PPCPs in each cluster based on the Abraham descriptors and log *K*_ow_ values and (4) validation using cross validation and statistical testing. The workflow is shown in Fig. 1.

### 2.1 Data collection

#### 2.1.1 Facilities and sample collection

Two full-scale facilities employing activated sludge wastewater treatment followed by distinct water reuse treatments were the subject of this study. A non-potable treatment plant employed denitrification-filtration/chlorination prior to distribution of reused water primarily used for irrigation. An indirect potable reuse plant employed advanced water treatment (FlocSed/Ozone/BAC/GAC/UV) prior to aquifer recharge. In total, 84 samples were collected from the two treatment facilities between November 2018 and August 2019, as described in a companion study focused on the fate of antibiotic resistant bacteria and antibiotic resistance genes.^45^

#### 2.1.2 PPCP multi-compound screening

All samples were processed immediately upon receipt and underwent pre-filtration using 0.7 μm glass fiber filters (Whatman, Maidstone, UK) and subdivided into 200 mL triplicates for analysis. As detailed in Section S1 of the ESI,† analytes were extracted, background matrices were cleaned up, and the final concentrates were obtained through solid-phase extraction (SPE). The resulting extracts were screened for the presence of 149 PPCPs, metabolites, dietary substances, agricultural chemicals, illegal drugs, and drug-testing agents that are commonly encountered in wastewater using UPLC/MS/MS (broadly referred to as “PPCPs” in this study), employing a semi-quantitative approach with a custom-made compound identification database.^46,47^ For the SPE, Oasis HLB cartridges from Waters with a 60 mg sorbent bed mass and 3 mL reservoir volume were used. The cartridges were pre-conditioned with 3 mL HPLC-grade methanol and 3 mL ultra-pure water. Subsequently, samples were processed through the cartridges at 5 mL min^−1^, and analytes were eluted with 3 mL HPLC-grade methanol, dried under N_2_ gas on a vacuum evaporation system (Labconco Kansas City, MO), and reconstituted with 1 mL HPLC-grade acetonitrile–water solution (1 : 1, v : v). UPLC-MS/MS was conducted using a 1290 UPLC/Agilent 6490 Triple Quad tandem MS (Agilent Technologies Inc., Santa Clara, CA). All samples were processed, cleaned, and analyzed within a single analytical batch to support comparisons of relative differences by comparing peak areas across samples.

#### 2.1.3 Abraham descriptors and log *K*_ow_ values

Abraham descriptors are parameters used to quantify key properties of a given chemical that govern its amenability to solvation and sorption. These descriptors include *E* (polarizability), *S* (dipolarity), *A* and *B* (hydrogen bond donating and accepting potential), *V* (molecular volume), and *L* (gas–hexadecane partition coefficient) (Table 1). Together, these parameters characterize the solvation properties of a compound: *E* and *S* relate to cavitation and van der Waals interactions; *A* and *B* characterize solute–solvent hydrogen bonding; *V* determines molecular size compatibility with substrate gaps; and *L* depicts bulk transport.

**Table 1:** Abraham descriptors for PPCP analysis

S no.	Symbol	Chemical characteristic	Units
1	*E*	Excess molar refraction	cm^3^ mol^−1^/10
2	*S*	Dipolarity/polarizability	Dimensionless
3	*A*	Hydrogen bonding acidity	Dimensionless
4	*B*	Hydrogen bonding basicity	Dimensionless
5	*V*	McGowan characteristic volume	cm^3^ mol^−1^/100
6	*L*	Gas-to-hexadecane partition	Dimensionless

We obtained the six Abraham descriptors for each PPCP from the publicly-available Helmholtz Centre for Environmental Research-Linear Solvation Energy Relationship (UFZ-LSER) database.^48^ This database provides experimental Abraham descriptors for most compounds using compound names, CAS-RN, or SMILES, and also offers calculated descriptors using only the SMILES of a compound. To complement this data, we obtained log *K*_ow_ values, which reflect hydrophobicity, from the publicly-available Environmental Protection Agency-Estimation Programs Interface (EPA-ESPI) Suite program.^49^ This comprehensive set of molecular descriptors enabled systematic characterization of PPCPs.

### 2.2 Clustering PPCPs based on relative abundance measures obtained from non-target screening

To classify PPCPs according to similarities in their removal patterns along the two treatment trains, we applied two distinct clustering approaches. The first approach was to group PPCPs as a function of which specific treatment process (*e.g.*, activated sludge, BAC, GAC, chlorination) achieved the greatest removal efficiency relative to the PPCP concentration measured in the influent. The second method was to cluster the PPCPs based on the removal pattern across each process relative to the influent to that process (*i.e.*, did the relative abundance increase or decrease relative to the previous treatment step?). These clustering methods are subsequently denoted as ‘C1’, and ‘C2’, respectively. Clustering analysis was applied to each individual treatment facility dataset. For preprocessing, PPCPs with missing relative abundance values across all treatment processes were removed and the common PPCPs shared among all events were extracted.

#### 2.2.1 C1: clustering based on the most efficient individual process relative to treatment train influent

The Unit Removal Efficiency (URE) for each PPCP was calculated using eqn (1):1
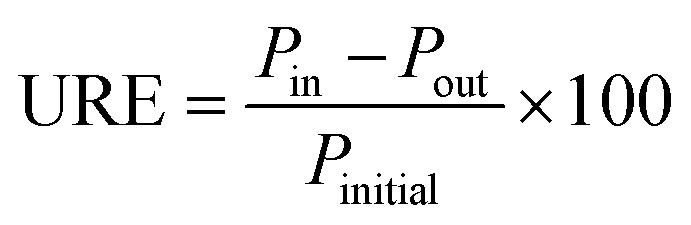
where *P*_in_ in represents the input peak area of a PPCP for treatment process *x*, *P*_out_ the output peak area of a PPCP for treatment process *x*, and *P*_initial_ is the input peak area of a PPCP in the wastewater influent at each facility. To obtain the representative URE across the four sampling events, for each treatment process, the average of the efficiencies from all events were calculated, where calculations comparing two subsequent below detection measurements were excluded. For each PPCP, the treatment process achieving the highest average initial removal efficiency was selected, and PPCPs sharing this same treatment process were clustered together.

#### 2.2.2 C2: clustering based on the removal pattern of PPCP in a given process relative to the immediately upstream process

The removal efficiency (RE) of each treatment process for each PPCP was calculated following eqn (2):2
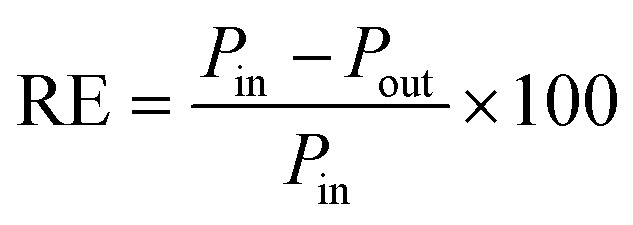
where *P*_in_ and *P*_out_ are the input and the output peak area of a compound of a treatment process, respectively. The average removal efficiency was determined across the four sampling trips for each treatment process. The average for each treatment process was then compared to that of the previous treatment process and transformed to one of four categorical variables: increase, decrease, same, or below detection (B.D.), resulting in a sequence of categorical variables for each PPCP, representing the overall removal pattern. Then, *K*-modes clustering^50^ which is specifically designed for categorical data, was applied to group PPCPs bearing similar removal patterns into clusters, where the *K* was set as 3.

### 2.3 Classification of PPCPs using Abraham descriptors and log *K*_ow_

PPCPs defined by C1 and C2 were further classified sing the Abraham descriptors and log *K*_ow_ values as inputs. Of particular interest was whether the PPCPs in the same cluster shared similar chemical characteristics. For this purpose, three machine learning-based algorithms were applied: support vector machine (SVM),^51^ random forest (RF),^52^ and logistic regression (LR),^53^ implemented based on ‘Scikit-learn’ package^54^ with the default hyperparameter settings. SVM is a supervised learning algorithm that identifies a hyperplane to create a decision boundary classifying the data points to each class by maximizing the margin between the classes. RF constructs multiple tree-structured classification models based on the set of discrete rules from the training dataset and aggregates the output from multiple trees to derive a final prediction output. LR estimates the probability for the given class using a sigmoid function to the output of linear regression function. We selected these three models due to their strong classification performance compared to methods like Naive Bayes and Decision Trees.^55^

### 2.4 Validation of the ML-based framework to predict the removal of PPCPs movement

To evaluate the proposed ML-based computational framework, several validation experiments were performed. First, we compared the clustering results obtained from C1 and C2 approaches between the two treatment facilities. Then, 5-fold cross validation was performed on the classification model to test the average accuracy, which was used as a metric to estimate whether the PPCPs can be predicted to one of the defined clusters based on the chemical properties. The PPCPs were divided into five parts (“folds”), with each part containing an equal number of PPCPs. In each iteration, four out of the five parts were used for training the ML-based classification models and the remaining part was solely used for testing. This process was repeated five times, with each fold serving as the test set exactly once. This evaluation followed standard cross validation approach^55–57^ and served to demonstrate whether the model can accurately predict the remove of new PPCPs that were not included in the training. Additionally, we performed *K*-mean clustering to group PPCPs based on the Abraham descriptor with log *K*_ow_ values to obtain the same number of clusters from C1 and C2. Based on the latter, we checked whether there was agreement between the PPCP clustering results using chemical properties and the clustering results based on the relative abundance using our proposed methods to determine whether the PPCPs in the same cluster share similar chemical properties. The overlap between PPCPs and the number/percentage of PPCPs in clusters defined by chemical properties *versus* those defined by remove efficiencies/patterns in the C1/C2 clustering approaches were compared.

### 2.5 Statistical analysis of the distinct chemical properties across PPCP clusters

Statistical testing was performed to assess whether the identified PPCP clusters showed distinct distributions of chemical properties across clusters, and similar properties within clusters. Using the Abraham descriptors and log *K*_ow_ values of each PPCP, Kruskal–Wallis *H*-test was performed by the basic statistic package in R (v4.1.2). Statistical significance was set at *p*-value <0.05. A user manual for running the proposed framework is provided in ESI† S2.

## 3. Results and discussion

### 3.1 Occurrence and removal of PPCPs across the two treatment trains

**Fig. 2:**
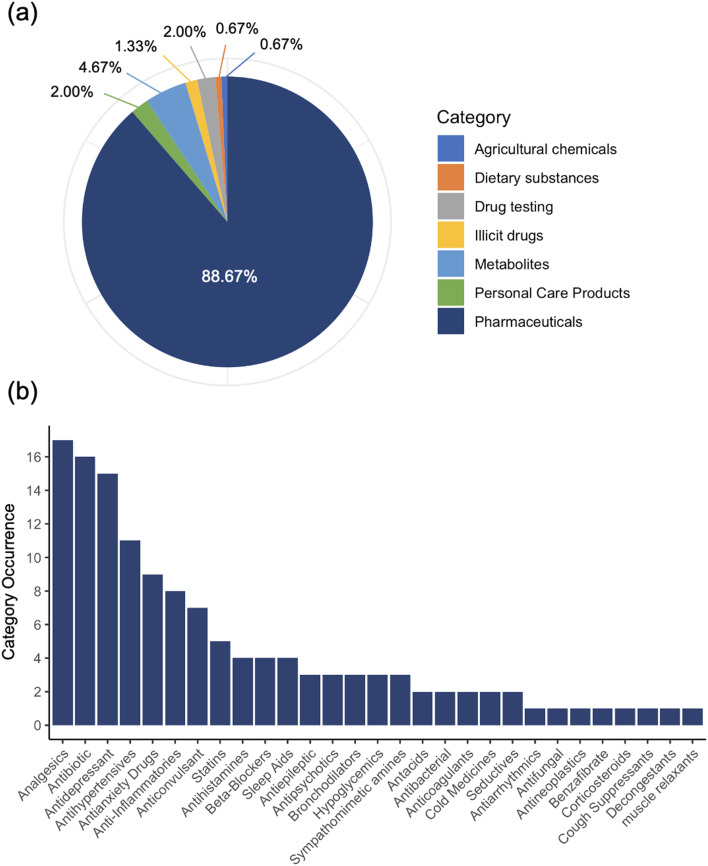
(a) Distribution of PPCPs across the two wastewater and water reuse treatment trains (b) frequency of occurrence of various categories in the pharmaceutical group across all samples (*n* = 84) collected from the various stages of treatment for the two treatment trains and across four sampling events.

Analysis of 149 PPCPs across two distinct treatment trains revealed a complex landscape of contaminant behavior and removal efficiencies, providing a rich dataset for developing and validating the PPCP removal prediction approach. Pharmaceuticals dominated the detected compounds (88.6%), with analgesics, antibiotics, and antidepressants showing the highest prevalence (Fig. 2b). The diverse array of compounds, including various therapeutic classes, pharmaceutical metabolites (4.67%), and personal care products (2%), presented a wide range of physicochemical properties, which supported development of robust predictive models.

The two treatment trains demonstrated varying levels of PPCP removal, with the potable reuse system achieving approximately 20% elimination of the screened compounds. The non-potable reuse system exhibited a 10% reduction. This is consistent with expectation of the advanced treatment processes employed in the potable reuse system, such as ozonation and UV, achieving greater overall removal.^58,59^

General patterns of PPCPs removal were consistent with expectation based on molecular structure. For instance, tramadol, is a complex organic compound that includes a tertiary amine and multiple aromatic rings and was found to be particularly recalcitrant. In contrast, chemicals like caffeine and acetaminophen were effectively eliminated, likely through sorption to solids. These observations underscore the potential of molecular descriptors, such as Abraham descriptors and log *K*_ow_ values, to capture nuanced relationships between chemical properties and efficacy of treatment.

The varying removal efficiencies observed across different treatment stages and between the two systems illuminated complex interplays between PPCP chemical properties and process-specific removal mechanisms. Hydrophobic compounds, exemplified by the anti-epileptic drug carbamazepine, generally persisted through initial treatment stages, but were successfully removed by ozone. Carbamazepine contains an electron-donating amine, which is known to be susceptible to ozonation, illustrating an expected linkage between molecular properties (*i.e.*, containing an electron-donating subgroup) and its susceptibility to treatment (*i.e.*, an electron-attracting oxidative process). Such results are consistent with prior studies that demonstrated the effectiveness of advanced oxidation processes, like ozonation and UV light, in eliminating hydrophobic and other electron-rich PPCPs.^60^

The heterogeneity in PPCP composition and removal patterns observed in this study not only illustrate the challenges of removing them *via* a unified treatment approach, but also highlights the potential for data-driven, predictive models to revolutionize treatment strategy optimization. By capturing nuanced relationships between molecular properties and treatment efficacies, ML models present a promising approach to enhance the ability to predict the fate of PPCPs across various treatment scenarios.

### 3.2 Quantifying PPCP removal patterns across treatment stages

**Fig. 3:**
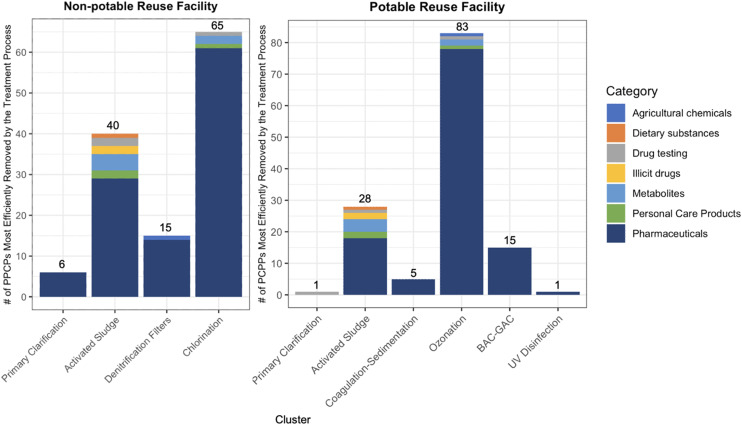
The number of PPCPs in each cluster based on the C1 clustering method, *i.e.*, according to the most efficient individual treatment process contributing to removal of each PPCP across the treatment train.

The C1 clustering approach revealed the relative contribution of each treatment stage to PPCP removal (Fig. 3). Oxidative processes formed the largest clusters, with ozonation being the most efficient removal method for 55.3% of the studied PPCPs in the potable reuse system. Chlorination was the primary removal mechanism for 43.3% of PPCPs in the non-potable system. This aligns with previous research highlighting the effectiveness of oxidative processes in degrading a wide range of organic contaminants.^61^

The activated sludge process also played a substantial role, being the most efficient removal stage for 18.7% and 26.6% of PPCPs in potable and non-potable systems respectively. This underscores the importance of biological processes in PPCP degradation,^62^ particularly for compounds susceptible to biodegradation. Physical processes, like primary clarification, formed smaller clusters, being the primary removal mechanism for only 4% of PPCPs in the non-potable system and 2% in the potable system. However, these processes still contributed to overall removal, with an average removal efficiency of 15% across all PPCPs. The PPCP lists for each cluster are detailed in Table S1† and the distribution of the removal efficiencies across treatment processes for each cluster are shown in Fig. S1.†

**Fig. 4:**
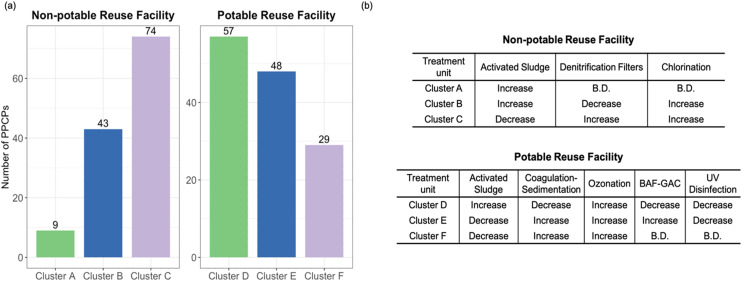
(a) The number of PPCPs in each cluster based on the C2 clustering approach, *i.e.*, removal pattern across the water reuse trains. Three clustering patterns were observed at each water reuse facility: ‘cluster A’, ‘cluster B’, and ‘cluster C’ for non-potable reuse facility and ‘cluster D’, ‘cluster E’, and ‘cluster F’ for potable reuse facility, where the removal patterns refer to changes in peak area relative abundance. (b) C2 PPCP removal patterns observed for the nonpotable and potable reuse facilities. B.D. – the compound was below detection by the time it reached the corresponding treatment stage. An “increase” and “decrease” were defined to represent changes in the representative removal efficiency of each treatment process, indicating an increase or decrease relative to the efficiency of the previous treatment stage, respectively.

The C2 clustering approach (Fig. 4, Table S2†) provided insight into the cumulative effects of each treatment stage. This analysis revealed that 68% of PPCPs experienced over 90% removal in the latter stages of treatment, particularly during oxidative processes. However, 22% of compounds showed substantial removal (>50%) in earlier stages, highlighting the importance of multi-barrier approaches in wastewater and reuse treatment trains.

It's important to note that removal efficiencies varied considerably among different PPCPs, even within the same treatment stage. For instance, while ozonation showed high removal efficiency (>90%) for 62% of PPCPs, it was less effective (<30% removal) for 18% of compounds. This variability underscores how inherent differences in PPCP physicochemical properties dictate the need for distinct treatment strategies.

These findings provide valuable insights into the relative contributions of different treatment stages to PPCP removal in wastewater and water reuse treatment trains. They highlight the importance of oxidative processes, such as ozone, while also demonstrating the significant role of biological treatment. Furthermore, they emphasize the value of a multi-barrier approach in achieving comprehensive PPCP removal, with each stage contributing to the overall reduction of PPCPs.

### 3.3 Predicting the removal of PPCPs through water reuse treatment trains using the ML-based classifiers

To predict PPCP removal patterns, we implemented ML-based classification models using Abraham descriptors and log *K*_ow_ values as inputs to independently classify each PPCP according to the clusters defined by the C1 and C2 approaches. We employed a five-fold cross-validation method using datasets from each treatment train, measuring classification accuracy on the test dataset to evaluate the models' ability to predict removal patterns for new, unseen PPCPs.

**Fig. 5:**
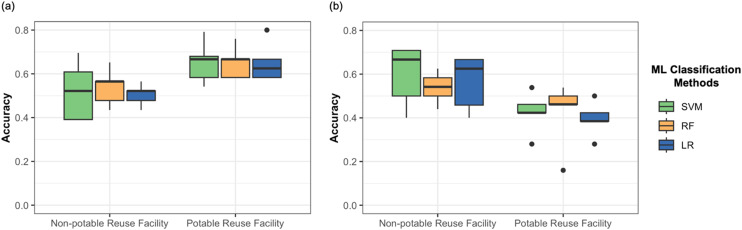
Classification performance results for predicting PPCPs to the clusters defined based on (a) C1 clustering and (b) C2 clustering approach for each treatment train, performing 5-fold cross validation. 5-Fold cross validation involves dividing the dataset into five subsets, training the model on four of them, and evaluating its performance on the fifth in a cyclic fashion, repeating this process five times to obtain a robust performance estimate. Machine learning-based (ML) classification methods: SVM – support vector machine; RF – random forest; LR – logistic regression. Each boxplot shows the distribution of accuracies for the ML classifiers in 5-fold cross validation of each cluster prediction, where the dot denotes the accuracies detected as the outlier by IQR rule.

For the prediction of PPCPs to clusters based on the C1 clustering approach (most efficient individual treatment process), random forest (RF) was found to achieve the highest average classification accuracies of 0.539 and 0.652 for the non-potable and potable reuse facilities, respectively (Fig. 5a). Support vector machine (SVM) showed similar performance with average accuracies of 0.522 and 0.652, while logistic regression (LR) yielded accuracies of 0.504 and 0.652 for the respective facilities. These results suggest that Abraham descriptors and log *K*_ow_ values capture significant information about a PPCP's susceptibility to specific treatment processes.

In classifying PPCPs according to the C2 clustering approach (removal pattern across all processes), SVM demonstrated the highest average accuracy of 0.597 for the non-potable facility, while achieving 0.425 for the potable facility (Fig. 5b). The second-best performances were observed with LR (0.563) for the non-potable facility and RF (0.424) for the potable facility. This variation in model performance between C1 and C2 classifications highlights complex relationships between molecular properties and overall removal patterns across multiple treatment stages. Additionally, using the random forest classifier, we measured the relative feature importance scores to identify which Abraham descriptors contributed most to predicting PPCP in each cluster for both C1 and C2. The results showed that, in most cases, ‘log *K*_ow_’ and ‘*V*’ were the two most important features for the prediction task. The exception was for predicting PPCPs into C2-based clusters in the potable reuse facility, where ‘*E*’ and ‘log *K*_ow_’ were the most significant features.

The approach developed here represents a significant advance in the application of ML techniques to PPCP fate prediction in water reuse systems. While a few studies have used ML for specific aspects of PPCP treatment, such as metal–organic frameworks removal capacity^63^ or photocatalytic degradation,^64^ our approach is the first to comprehensively characterize PPCP removal across various water reuse treatment processes using molecular descriptors.

The accuracies achieved by our ML models ranged from 42.5% to 65.2% and depended on the facility and clustering approach. While accuracy was moderate, it was not unexpected considering the complex nature of PPCP removal processes and the limited previous applications of ML in this domain. Overall, these results demonstrate the potential of the ML framework to predict PPCP removal patterns based solely on Abraham descriptors and log *K*_ow_ values, which can be improved upon in the future. The findings further support the hypothesis that PPCP physicochemical properties can predict PPCP response to various treatment processes.

The variation in model performance between C1 and C2 classifications and between facilities suggests that different ML algorithms may be more suitable for specific aspects of PPCP removal prediction. This underscores the value of our multi-model approach in capturing diverse aspects of PPCP behavior in water reuse systems, aligning with our aim to identify underlying associations between physicochemical properties and removal patterns.

Furthermore, the ability of our models to achieve good accuracy using only Abraham descriptors and log *K*_ow_ as inputs is particularly noteworthy. It suggests that molecular properties, specifically, and physicochemical properties generally, are indeed relevant predictors of PPCP fate in water reuse treatment processes, validating our approach of using these descriptors to classify PPCPs according to their defined clusters.

The findings of this study not only advance the application of ML in PPCP removal prediction, but also provide a foundation for future refinements and expansions of this approach. By demonstrating the feasibility of using ML to characterize PPCP removal in wastewater and water reuse facilities based on molecular descriptors and other physicochemical properties, our study opens new avenues for optimizing treatment processes and assessing the fate of emerging contaminants.

### 3.4 Validation of distinct physicochemical properties across PPCP clusters

In addition to the 5-fold cross validation in section 3.3, we investigated whether the PPCPs in each cluster shared similar physicochemical properties and distinct distributions compared to other clusters. Abraham descriptor and log *K*_ow_ values are compared by cluster in Fig. S2 and S3.† It was found that, Abraham descriptors *A* and *E* were significantly different across the C1 clusters for both treatment facilities (Kruskal–Wallis, *p*-value <0.05). However, no significant difference in these values was found across the C2 clusters (Fig. S4†).

**Fig. 6:**
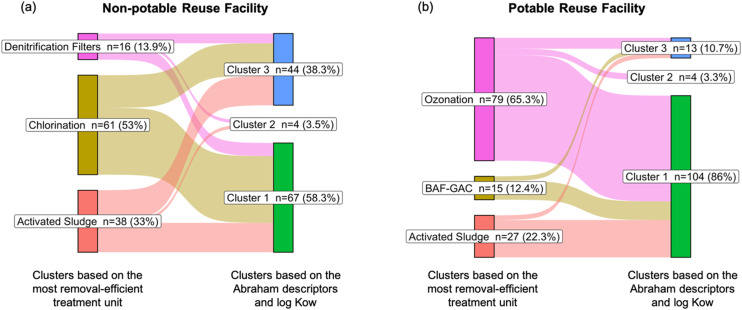
Number of PPCPs overlapping among the clusters based on C1 clustering, *i.e.*, the treatment process that achieved the most efficient removal, and based on the physicochemical properties for (a) non-potable and (b) potable reuse facility.

**Fig. 7:**
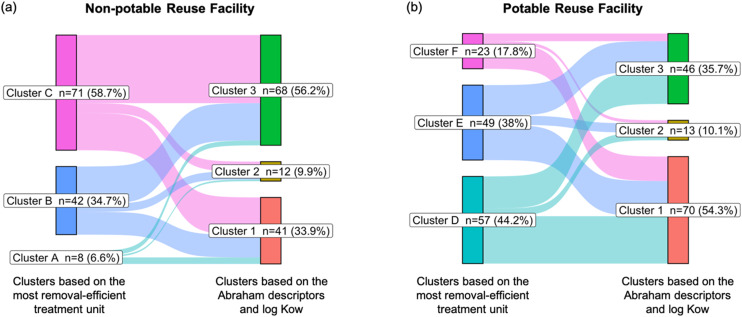
Number of PPCPs overlapping among the C2 clusters, based on the removal pattern across the facility, and clusters based on the physicochemical properties for (a) non-potable and (b) potable reuse facility.

We further performed *K*-mean clustering to group PPCPs based on the Abraham descriptor and log *K*_ow_ values and checked whether there was agreement between the PPCP clustering results using physicochemical properties *versus* removal efficiencies/patterns defined by the C1/C2 clustering approaches. It was found that 61 and 71 PPCPs overlapped in their groupings based on C1 clustering at the non-potable and potable treatment facilities, respectively (Fig. 6). For the C2 clustering approach, 56 and 54 PPCPs overlapped in their classification based on the Abraham descriptor and log *K*_ow_ values (Fig. 7). The full list of overlapping PPCPs is reported in Tables S4 and S5.†

Among the C1 clustering overlaps, PPCPs were not dominated by a single category, but represented a diverse range of pharmaceuticals. However, analgesics, antidepressants, antianxiety drugs, and antihypertensives were more prevalent among the overlapping clusters (Table S6†). These compounds predominantly clustered under oxidative processes, *i.e.*, ozonation for potable reuse systems and chlorination for non-potable reuse systems. Upon further analysis of Abraham descriptors represented by these clusters (Fig. S2†), we found that PPCPs in these clusters exhibited higher *A*, *B* (hydrogen bond basicity), and log *K*_ow_ values compared to other clusters. This indicates a trend of higher hydrophobicity, which supports more effective removal through advanced oxidation processes such as ozonation and chlorination.^65^

Among the C2 clustering, we observed a comparable dominance of the PPCP categories in the overlapping clusters. Most of these PPCPs were found in cluster C for non-potable systems and clusters D and E for potable systems. In terms of treatment trends, chlorination (for non-potable systems) and ozonation (for potable systems) again achieved greater percent removal compared to earlier treatment stages. Further analysis of Abraham descriptors for these clusters (Fig. S3†) revealed similar trends in chemical properties, with PPCPs in these clusters having higher *A*, *B*, and log *K*_ow_ values. This further suggests that higher hydrophobicity and these specific chemical properties contribute to the enhanced removal efficiency observed in both ozonation and chlorination processes.^65^

## 4. Future directions

This study demonstrates a promising avenue to predict the removal of emerging, previously uncharacterized, PPCPs through various candidate process treatment processes, based on their physicochemical properties. This could be a valuable approach towards treatment train design and operation for maximal removal. While promising, further refinement and testing of the approach developed herein would be beneficial. To move towards developing more accurate predictive models for PPCP removal, expanding available databases summarizing key PPCP physicochemical parameters would be of value, including biodegradation kinetics, reaction rate constants, chemical structures, and sorption coefficients. Incorporating localized temporal variation in environmental conditions such as temperature, pH, or rainfall could also help account for variability in concentration trends. Changes in such factors are known to influence contaminant degradation and transport mechanisms.^66^ Furthermore, operational parameters such as differing dissolved oxygen concentrations, solids retention times, and flowrates used across different facilities could explain some of the variance observed between facilities. With these data resources in place, advanced deep learning algorithms capable of capturing nonlinear relationships, such as multi-layer artificial neural networks can be implemented to relate PPCP properties and influent concentrations to effluent concentrations and removal efficiencies across treatment steps.

Emerging generative modeling techniques could be one avenue for overcoming key data limitations. Following model development, further validation using data from additional treatment trains not included in the training data set would be of value. Once validated, user-friendly tools could be developed for consultants, plant operators and regulators. By inputting PPCP properties and operating conditions, the models could efficiently predict expected removal and guide the design and operation of corresponding treatment trains. Applications could include risk assessment of new chemicals and *in silico* screening prior to market entry. Overall, leveraging ML on expanded PPCP data has potential to enable predictive approaches that bolster wastewater and water reuse treatment and management strategies.

## 5. Limitations of sampling approach and considerations

This study provides valuable insights into the observed differences between influent and effluent compositions across and advanced water treatment train. Sampling was conducted over four events in order to capture general removal trends over time. However, ideally, sampling could have been more precisely timed to account for hydraulic retention time (HRT) and to attempt to follow the same parcel of water through each unit treatment process. Considering this limitation, the term ‘removals’ used throughout the manuscript should be interpreted as indicative of typical differences in influent *versus* effluent during stable operation, rather than as definitive removal efficiencies. Future studies could consider timing sampling in a manner that takes into account HRT as a means to account for temporal variation in treatment dynamics and enhance the precision of removal estimates.

## 6. Conclusion

In this study, 149 PPCPs were screened through wastewater treatment and subsequent potable and non-potable water reuse treatment trains using non-targeted UPLC-MS/MS analysis to evaluate efficacy of various physical, biological, oxidative, and sorptive treatment processes for their removal. PPCPs were clustered based on the relative abundances measured through each treatment step using two approaches: C1 grouped PPCPs based on their most efficient individual treatment process, while C2 clustered PPCPs according to their removal pattern across the treatment train. ML-based classification algorithms including SVM, RF, and LR were applied to relate PPCP physicochemical descriptors to their cluster assignments. The results suggested that PPCPs within each cluster generally share similar physicochemical properties, as reflected by similarities among Abraham descriptors *E*, *S*, *A*, *B*, and *V*. Further, each cluster has distinct characteristics from one another. The C1 clustering provides insight into the most suitable treatment technology for specific PPCPs. Meanwhile, the C2 clustering elucidates general trends of PPCP persistence and removal in reuse systems.

Here, a novel framework for predicting PPCP removal by various treatment processes was developed combining supervised and unsupervised ML and informed by specific physicochemical properties of each PPCP. This study demonstrated the ability of ML techniques; RF, SVM and LR, to systematically characterize and classify PPCP removal, using extensive PPCP screening data sets collected through two wastewater treatment plants followed by distinct water reuse treatment trains. Looking forward, considering additional molecular descriptors, and utilizing more advanced ML techniques and drawing from a broader array of data sets can help to further develop this framework into a practical, accurate tool for consultants, operators and regulators. The framework developed here could be of particular value for informing the design of water reuse treatment trains to meet ever growing demands for removal of a broader array of PPCPs, including emerging contaminants of concern. The approach could help to complement and amplify the value of costly direct testing and monitoring of PPCPs in wastewater and reuse treatment trains.

## Data availability

The complete framework of the code used in this study is provided in ESI† S2. All major data related to this study are reported in the supplementary tables and figures. Additionally, the complete dataset can be accessed at https://github.com/joungmin-choi/ML-PPCP.

## Author contributions

Development and implementation of the data analysis approach were performed by JMC and VM. Sampling strategy and collection was performed by IK, who also assisted with preliminary analysis of the data. CC performed the PPCP analysis under supervision of KX. The first draft of the manuscript was written by JMC and VM. AP, LZ, KX, PJV, MFB, CB and CB contributed to conceptualization of the study, supervision, review and editing. All authors read and approved the final manuscript.

## Conflicts of interest

Other than funding provided by the employer of co-author Charles Bott, the authors declare that they have no known competing financial interests or personal relationships that could have appeared to influence the work reported in this paper.

## Supplementary Material

EW-011-D4EW00892H-s001

EW-011-D4EW00892H-s002

EW-011-D4EW00892H-s003
