# Supplementary material for: A machine learning framework to predict PPCP removal through various wastewater and water reuse treatment trains
Source: Environ Sci (Camb). 2024 Dec 19;11(2):481–93. doi: 10.1039/d4ew00892h (PMC11694563; doi:10.1039/d4ew00892h)
Supplement: EW-011-D4EW00892H-s001 [file EW-011-D4EW00892H-s001.pdf]

Supplementary Information (SI) for Environmental Science: Water Research & Technology.
This journal is © The Royal Society of Chemistry 2024

Supplementary Figures

Supplementary Figure S1 Distribution of the representative process treatment efficiencies across each treatment process among the PPCPs for each corresponding C1 cluster. Min-max normalization with the range of -100 to 100 was performed for improved visualization. (a) Non-potable reuse and (b) potable reuse treatment trains

Supplementary Figure S2 Distribution of Abraham descriptors for each cluster based on the C1 clustering approach, i.e., the removal pattern across the facility in (a) non-potable and (b) potable reuse facility

Supplementary Figure S3. Distribution of Abraham descriptors for each cluster based on the C2 clustering approach, i.e., the removal pattern across the facility in (a) non-potable and (b) potable reuse facility

Supplementary Figure S4. Distribution of Abraham descriptors that were associated with statistically significant differences based on Kruskal-Wallis test (p-value < 0.05) across the clusters based on the C1 clustering method, i.e., the most efficient individual treatment process at each facility.

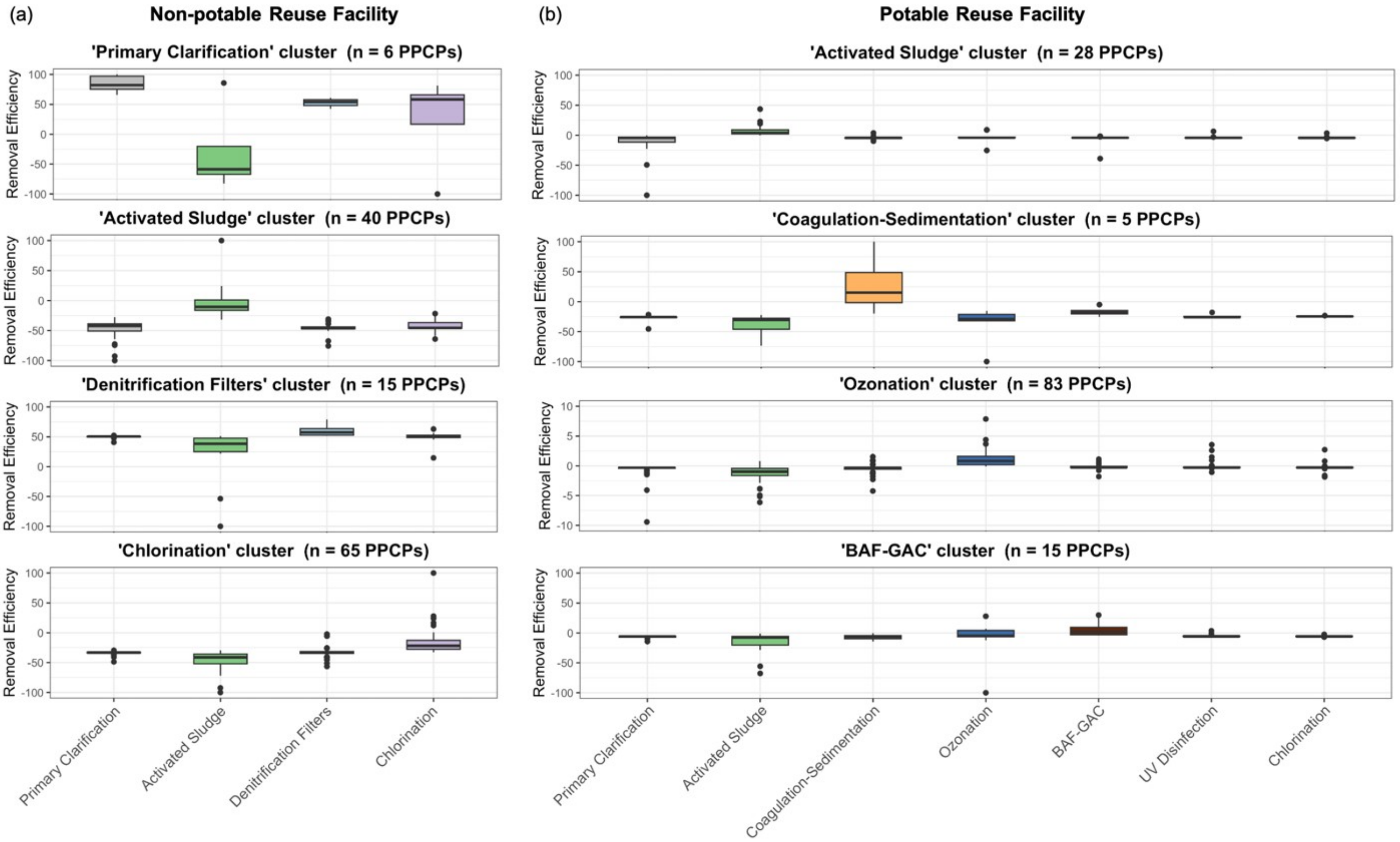

Supplementary Figure S1. Distribution of the representative process treatment efficiencies across each treatment process among the PPCPs for each corresponding C1 cluster. Min-max normalization with the range of -100 to 100 was performed for improved visualization. (a) Non-potable reuse and (b) potable reuse treatment trains.

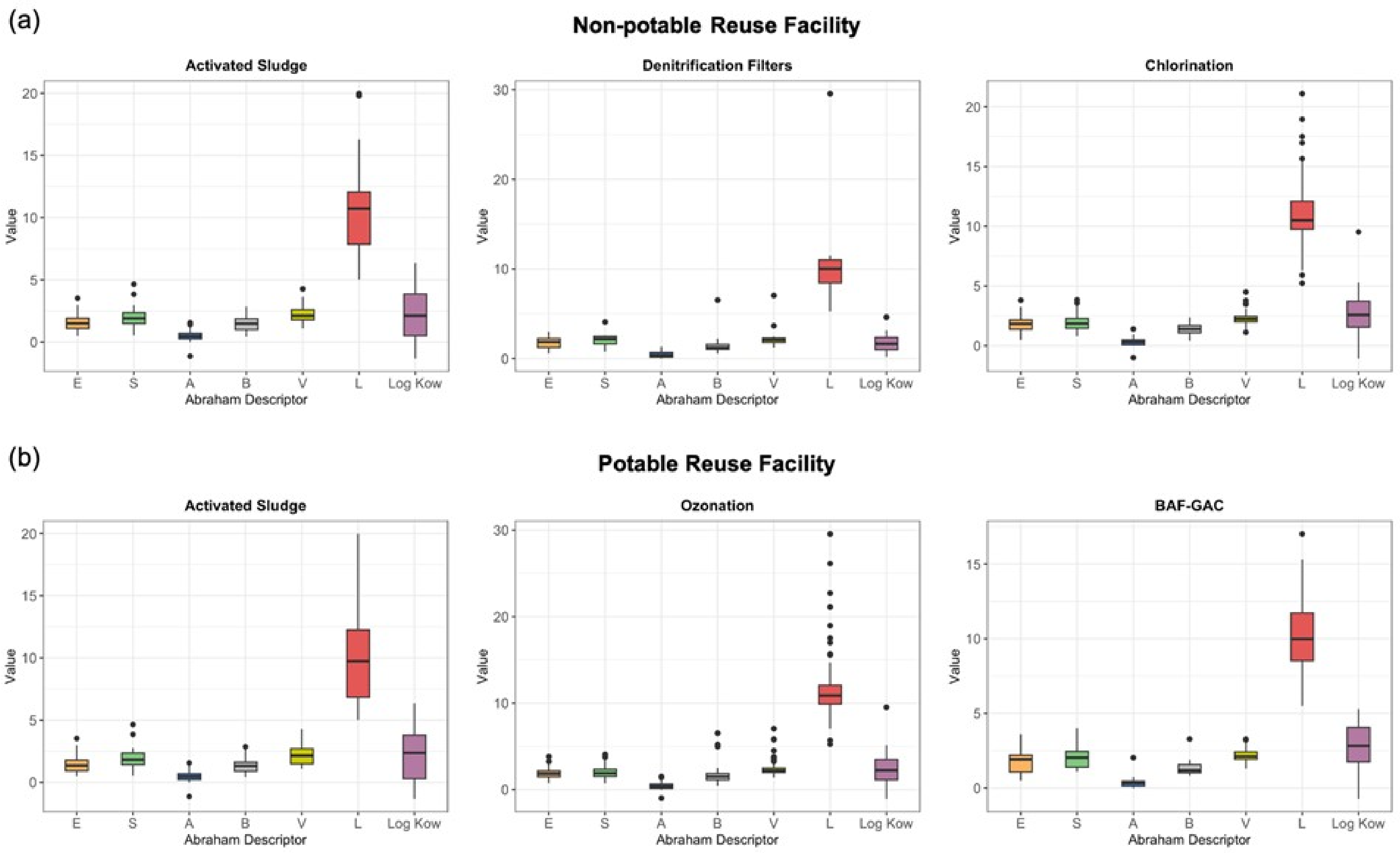

Supplementary Figure S2. Distribution of Abraham descriptors for each cluster based on C1 clusters, i.e., the most efficient individual treatment process for corresponding removal of the PPCP for (a) non-potable and (b) potable reuse facility

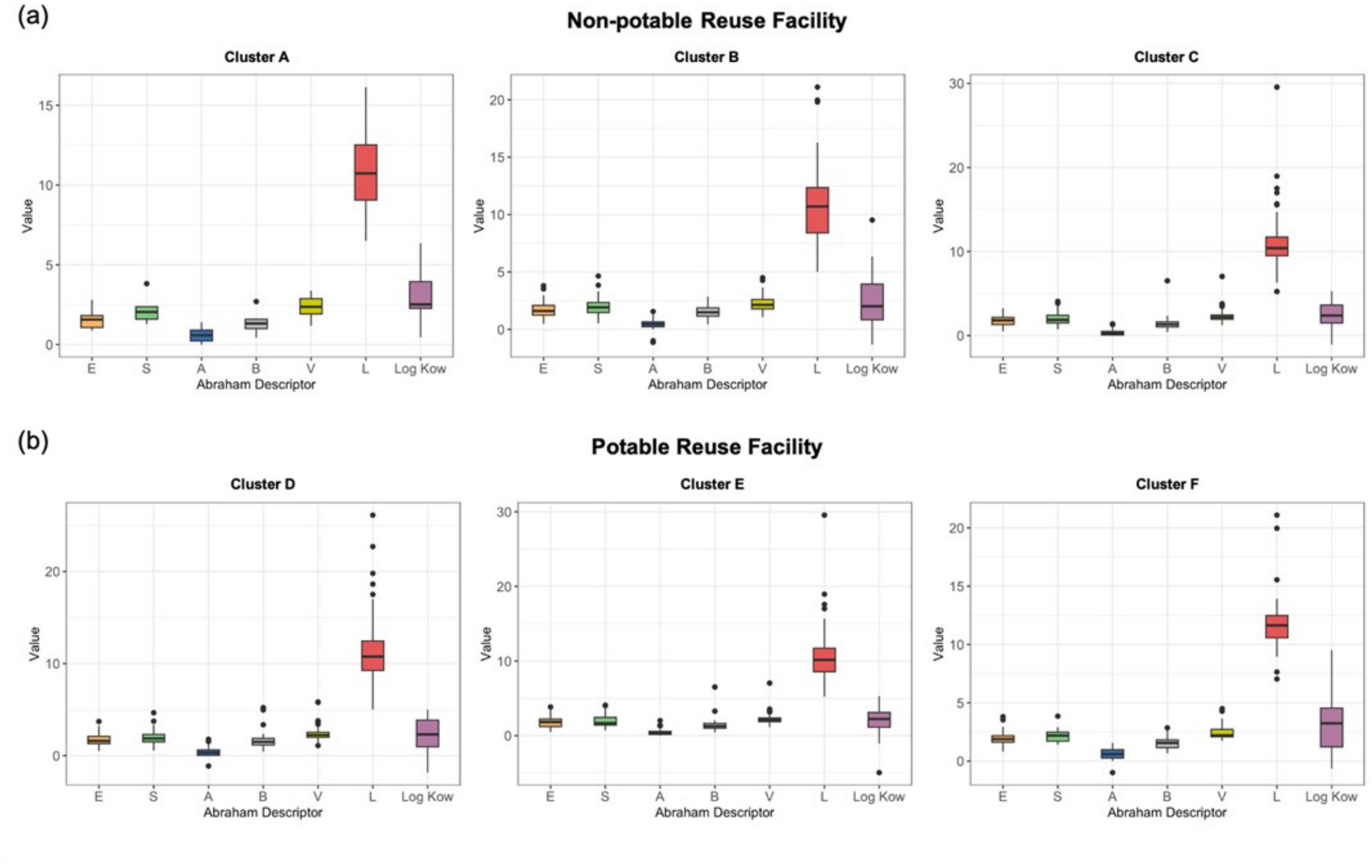

Supplementary Figure S3. Distribution of Abraham descriptors for each cluster based on the C2 clustering approach, i.e., the removal pattern across the facility in (a) non-potable and (b) potable reuse facility .

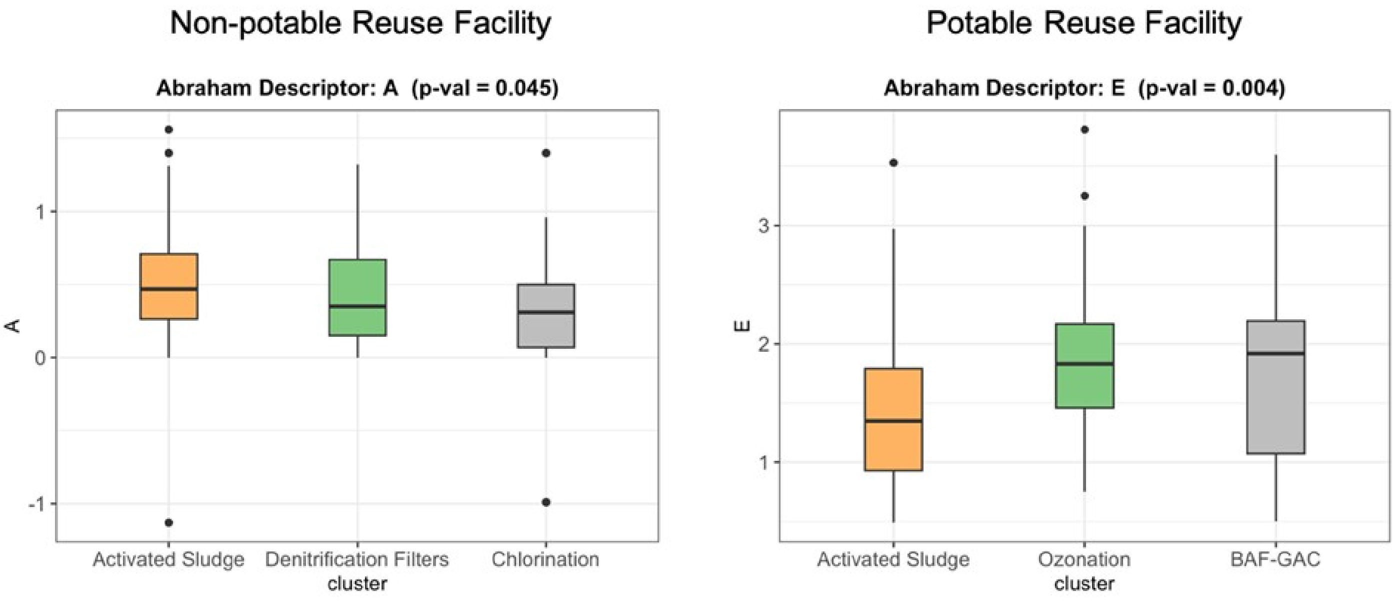

Supplementary Figure S4. Distribution of Abraham descriptors that were associated with statistically significant differences based on Kruskal-Wallis test (p-value < 0.05) across the clusters based on the C1 clustering method, i.e., the most efficient individual treatment process at each facility
