# Supplementary material for: A machine learning framework to predict PPCP removal through various wastewater and water reuse treatment trains
Source: Environ Sci (Camb). 2024 Dec 19;11(2):481–93. doi: 10.1039/d4ew00892h (PMC11694563; doi:10.1039/d4ew00892h)
Supplement: EW-011-D4EW00892H-s003 [file EW-011-D4EW00892H-s003.pdf]

Supplementary Information (SI) for Environmental Science: Water Research & Technology.
This journal is © The Royal Society of Chemistry 2024

Supplementary Information S1

Extraction and cleanup of water samples using solid phase extraction (SPE)

An aliquot of 200 mL water sample pre-filtered with a 0.7 µm 55 mm in diameter glass fiber filter (Whatman, Maidstone, UK) was loaded onto a SPE cartridge (hydrophilic-lipophilic balance cartridge, Waters, Milford, MA) that was pre-conditioned with 3 mL HPLC-grade methanol and 3 mL ultra-pure water. The sample flow through the cartridge was maintained at 5 mL min-1. Once the sample passed through the cartridge, the cartridge was then dried for 10 mins by gently pulling air through it to remove any remaining water. The PPCPs were then eluted off the cartridge with 3 mL HPLC-grade methanol. The eluted sample was then completely dried under N2 gas on a vacuum evaporation system (RapidVap, Labconco Kansas City, MO), reconstituted with LC-grade acetonitrile-water solution (1:1, v:v) to 1 mL, filtered through a 0.2 µm polytetrafluoroethylene (Thermo Scientific, Waltham, MA) syringe filter into a 2 mL glass amber vial (Agilent, Santa Clara, CA), and then screened for 150 PPCPs on a ultra-performance liquid chromatography-tandem mass spectrometry (UPLC/MS/MS) using method described below.

UPLC/MS/MS screening for PPCPs

An ultra-performance liquid chromatography (UPLC) (1290, Agilent Technology, Santa Clara, CA) coupled with a tandem mass spectrometry (6490, Agilent Technology, Santa Clara, CA) was used for the multi-compounds screening of 150 PPCPs. The PPCP screening method was developed based on a compounds database and chromatographic method that was custom-developed by the Agilent Technology upon analysis of 140 analytical standards of compounds which were reported to be the most detected in environmental water (Yang 2015). This database was developed using the same model of analytical column (Agilent Zorbax Extend C18 analytical column, 5 µm × 4.6 mm × 50 mm) and UPLC/MS/MS (Agilent 1290-6490) as that used in our study. This database contains specific compound identification criteria (i.e., retention time, precursor ions, product ions, collision energies) for the identification of each of the 150 PPCPs.

For the chromatographic section of the method, compounds were separated on the Agilent Zorbax Extend C18 analytical column (4.6 mm × 50 mm, 5 µm). The temperature of the analytical column oven was kept at 40°C. The mobile phase consisted of water with 0.1% formic acid (component A) and water with 95% acetonitrile (component B). The mobile phase flow rate was 0.3mL min-1 under a gradient elution of 0 - 15 min, 95% - 10% A, 15 - 17.5 min, 10%A, 17.5-18 min, 10% - 95%A, and 18 – 20 min, 95%A. The total sample injection volume was 20 µL.

For the mass spectrometry section of the method, electrospray ionization (ESI) and multiple reaction monitoring (MRM) mode was used. Each sample was analyzed separately in both positive and negative modes to detect oppositely charged ions. A compound in a sample was identified based on matching the masses of its precursor ion, product ion I, and product ion II

with those listed in the database and further confirmed based on matching chromatographic retention time and peak shape between product ion I and II. In addition, if the signal to noise ratio of the chromatographic peak of one of the product ions for a suspected compound was less than 3, the identity of this compound was rejected.

For quality assurance and quality control (QA/QC), one laboratory blank (ultrapure Milli-Q water) and one spiked recovery sample (ultrapure Milli-Q water spiked with known concentrations of four analytical standards: Carbamazepine, Erythromycin, Sulfamethazine, Chlorotetracycline, Tylosin, Sulfamethoxazole, and Triclosan) were extracted, cleaned up, concentrated, and analyzed along with the water samples for every 20 samples to monitor possible cross contamination of the compounds screened for and recovery of the spiked compounds. In addition, an instrument blank sample (UPLC mobile phase) was injected for every 20 samples to monitor possible instrument cross contamination. All analytes screened for, were not detectable in any of the instrumental blanks or laboratory blanks, indicating free of cross contamination of those compounds during sample processing and analysis.

Supplementary Information S2.
Tutorial for running a machine learning framework to predict PPCP removal through various water reuse treatment processes.

- Requirements
  - python (>= 3.11.3)
  - scikit-learn (>= 1.2.2)
  - pandas (>= 2.0.2)
  - numpy (>= 1.24.3)
  - kmodes (v0.12.2)

- Installation
  - To install the above requirements, please run below commands in the terminal:
    pip install numpy pandas os sys kmodes
    pip install -U scikit-learn

- Input
  1) Prepare a dataset with the relative abundance values of PPCPs across the treatment units in a data matrix, and save it in CSV file format
     - Each row should represent a PPCP, and each column should represent a treatment unit
     - The first row should contain the names of the treatment plants, and the first column should contain the names of the PPCPs.
     - Example : dataset.csv

PPCP	Primary Clarification	Activated Sludge	Ozonation	…
Acebutolol	8974	92351.6	6265.5	…
Atenolol	110756.2	19311	0	…
…	…	…	…	…

  2) Prepare a dataset with the Abraham descriptors of PPCPs, and save it in CSV file format
     - Each row should represent a PPCP, and each column should represent a Abraham descriptors
     - The first row should contain the names of the Abraham descriptors, and the first column should contain the names of the PPCPs.
     - Example : abraham_descriptors_dataset.csv

PPCP	E	S	A	…
Acebutolol	1.6	2.42	0.9	…
Atenolol	1.45	1.9	0.62	…
…	…	…	…	…

- Calculation of Unit Removal Efficiency (URE) for each PPCP
    - Run the below code using python3
    - Output : “results_URE.csv” having URE values for each PPCP

```
import pandas as pd
import numpy as np

filename = "dataset.csv" #Change to your dataset filename

data = pd.read_csv(filename)
data.dropna(axis = 0, inplace = True)
data.reset_index(inplace = True, drop = True)

ppcp_list = data['PPCP'].tolist()
treatment_stage_list = data.columns.tolist()
treatment_stage_list.remove('PPCP')

init_list = []
for i in range(len(treatment_stage_list)-1) :
	init_list.append(0)
	rm_eff_dict = {}
	count_dict = {}
	for ppcp in ppcp_list :
		rm_eff_dict[ppcp] = init_list.copy()
		count_dict[ppcp] = init_list.copy()

for i in range(len(data)) :
	for j in range(len(treatment_stage_list)-1) :
		tmp_stage_in = treatment_stage_list[j]
		tmp_stage_out = treatment_stage_list[j+1]

		tmp_ppcp = data['PPCP'][i]
		if (data[treatment_stage_list[0]][i] != 0) and (data[tmp_stage_in][i] != 0) :
			tmp_rm = (data[tmp_stage_in][i] - data[tmp_stage_out][i]) /
data[treatment_stage_list[0]][i] * 100
			if tmp_ppcp not in ppcp_list :
				rm_eff_dict[tmp_ppcp] = init_list.copy()
				count_dict[tmp_ppcp] = init_list.copy()
			rm_eff_dict[tmp_ppcp][j] += tmp_rm
			count_dict[tmp_ppcp][j] += 1

count_df = pd.DataFrame(count_dict)
rm_eff_df = pd.DataFrame(rm_eff_dict)

rm_eff_avg_df = rm_eff_df.copy()
```

```
final_ppcp_list = rm_eff_df.columns.tolist()
for ppcp in final_ppcp_list :
	for i in range(len(rm_eff_df)) :
		if count_df[ppcp][i] != 0 :
			rm_eff_avg_df[ppcp][i] = rm_eff_avg_df[ppcp][i]/count_df[ppcp][i]
		else :
			rm_eff_avg_df[ppcp][i] = np.nan

rm_eff_avg_df = rm_eff_avg_df.T
rm_eff_avg_df.dropna(how = 'all', inplace = True)
rm_eff_avg_df.to_csv("results_URE.csv", mode = "w", index = True)
```

- Calculation of Removal Efficiency (RE) for each PPCP
    - Run the below code using python3
    - Output : “results_RE.csv” having RE values for each PPCP

```
import pandas as pd
import numpy as np

filename = "dataset.csv" #Change to your dataset filename

data = pd.read_csv(filename)
data.dropna(axis = 0, inplace = True)
data.reset_index(inplace = True, drop = True)
ppcp_list = data['PPCP'].tolist()
treatment_stage_list = data.columns.tolist()
treatment_stage_list.remove('PPCP')
init_list = []

for i in range(len(treatment_stage_list)-1) :
	init_list.append(0)
	rm_eff_dict = {}
	count_dict = {}
	for ppcp in ppcp_list :
		rm_eff_dict[ppcp] = init_list.copy()
		count_dict[ppcp] = init_list.copy()
```

```
for i in range(len(data)) :
        for j in range(len(treatment_stage_list)-1) :
                tmp_stage_in = treatment_stage_list[j]
                tmp_stage_out = treatment_stage_list[j+1]

                tmp_ppcp = data['PPCP'][i]
                if data[tmp_stage_in][i] != 0 :
                        tmp_rm = (data[tmp_stage_in][i] - data[tmp_stage_out][i]) /
data[tmp_stage_in][i] * 100
                        if tmp_ppcp not in ppcp_list :
                                rm_eff_dict[tmp_ppcp] = init_list.copy()
                                count_dict[tmp_ppcp] = init_list.copy()
                        rm_eff_dict[tmp_ppcp][j] += tmp_rm
                        count_dict[tmp_ppcp][j] += 1


count_df = pd.DataFrame(count_dict)
rm_eff_df = pd.DataFrame(rm_eff_dict)

rm_eff_avg_df = rm_eff_df.copy()

final_ppcp_list = rm_eff_df.columns.tolist()
for ppcp in final_ppcp_list :
        for i in range(len(rm_eff_df)) :
                if count_df[ppcp][i] != 0 :
                        rm_eff_avg_df[ppcp][i] = rm_eff_avg_df[ppcp][i]/count_df[ppcp][i]
                else :
                        rm_eff_avg_df[ppcp][i] = np.nan

rm_eff_avg_df = rm_eff_avg_df.T
rm_eff_avg_df.dropna(how = 'all', inplace = True)
rm_eff_avg_df.to_csv("results_RE.csv", mode = "w", index = True)
```

- Run C1 clustering and perform 5-fold cross validation based on ML classifiers
    - Run the below code using python3
    - Output : “results_c1_cluster.csv” having C1 clustering results with “results_pred_acc.csv” having accuracy results for 5-fold cross validation based on ML classifiers

```
import pandas as pd
import os
from sklearn.model_selection import KFold
from sklearn import svm, linear_model
from sklearn.ensemble import RandomForestClassifier
from sklearn.linear_model import LogisticRegression
from sklearn.metrics import accuracy_score
from sklearn.metrics import f1_score

abraham_data_filename = 'abraham_descriptors_dataset.csv' #Change to your dataset
filename
filename = "results_URE.csv"

data = pd.read_csv(filename, index_col = 0)
data.dropna(inplace = True)

stage_list = data.columns.tolist()
data.reset_index(inplace = True)

cluster_list = []
cluster_num_list = []

for i in range(len(data)) : #len(data)
	tmp_abun = -1000.0
	tmp_stage = ''
	for stage in stage_list :
		if data[stage][i] > tmp_abun :
			tmp_abun = data[stage][i]
			tmp_stage = stage
	cluster_list.append(tmp_stage)
	cluster_num_list.append(stage_list.index(tmp_stage))

data["cluster"] = cluster_list
data["cluster_num"] = cluster_num_list
data.to_csv(resDir + wwtp_name + "_results.csv", mode = "w", index = False)
data[['PPCP', 'cluster', 'cluster_num']].to_csv("results_c1_cluster.csv", mode = "w", index =
False)
```

```
data = pd.read_csv("results_c1_cluster.csv", index_col = 0)
abraham_data = pd.read_csv(abraham_data_filename, index_col = 0)
tmp = scaler.fit_transform(abraham_data)
tmp = pd.DataFrame(tmp)
tmp.columns = abraham_data.columns
tmp.index = abraham_data.index
abraham_data = tmp
X = pd.merge(data, abraham_data, left_index = True, right_index = True)
del X['cluster']
X.groupby('cluster_num').count()
y = X['cluster_num'].values
del X['cluster_num']
X.reset_index(inplace = True, drop = True)
kf = KFold(n_splits=5, shuffle = True, random_state = 42)
svm_acc_list = []
rf_acc_list = []
lr_acc_list = []
i = 1
for train_index, test_index in kf.split(X):
    X_train, X_test = X.loc[train_index], X.loc[test_index]
    y_train, y_test = y[train_index], y[test_index]
    svm_classifier = svm.SVC(kernel = 'rbf', probability=True, random_state=42)
    svm_classifier = svm_classifier.fit(X_train, y_train)
    svm_predicted_class = svm_classifier.predict(X_test)
    tmp_acc = accuracy_score(y_test, svm_predicted_class)
    svm_acc_list.append(tmp_acc)
    rf_classifier = RandomForestClassifier(random_state=42)
    rf_classifier = rf_classifier.fit(X_train, y_train)
    rf_predicted_class = rf_classifier.predict(X_test)
    tmp_acc = accuracy_score(y_test, rf_predicted_class)
    rf_acc_list.append(tmp_acc)
    lr_classifier = LogisticRegression(random_state=42)
    lr_classifier = lr_classifier.fit(X_train, y_train)
    lr_predicted_class = lr_classifier.predict(X_test)
    tmp_acc = accuracy_score(y_test, lr_predicted_class)
    lr_acc_list.append(tmp_acc)
    i = i + 1

acc_res = pd.DataFrame({'SVM' : svm_acc_list, 'RF' : rf_acc_list, 'LR' : lr_acc_list})
acc_res.loc['avg'] = acc_res.mean()

acc_res.to_csv("results_pred_acc.csv", mode = "w", index = True)
```

- Run C2 clustering and perform 5-fold cross validation based on ML classifiers
    - Run the below code using python3
    - Output : “results_c2_cluster.csv” having C1 clustering results with “results_pred_acc.csv” having accuracy results measured for 5-fold cross validation based on ML classifiers

```
import pandas as pd
import os
from sklearn.cluster import KMeans
from kmodes.kmodes import KModes
abraham_data_filename = 'abraham_descriptors_dataset.csv' #Change to your dataset
filename
filename = "results_RE.csv"

data = pd.read_csv(filename, index_col = 0)
data.dropna(inplace = True)

stage_list = data.columns.tolist()
pattern_list = []
for i in range(len(stage_list)) :
        pattern_list.append([])

for i in range(len(data)) :
        for j in range(len(stage_list)) :
                if j == 0 :
                        control = data[stage_list[j]][i]
                else :
                        tmp_value = data[stage_list[j]][i]
                        if tmp_value == 0 :
                                pattern_list[j].append("B.D.")
                                control = tmp_value
                                continue
                        diff = tmp_value - control
                        if control == 0 :
                                diff_var = 100
                        else :
                                diff_var = diff/control * 100
                        if diff_var < 0 :
                                diff_var = diff_var * -1
                        #
                        if diff_var <= 10 :
                                pattern_list[j].append("same")
                        else :
                                if diff > 0 :
                                        pattern_list[j].append("up")
                                else :
                                        pattern_list[j].append("down")
                        control = tmp_value
```

```
ppcp_list = data.index.tolist()
tmp_dict = {}
tmp_dict["PPCP"] = ppcp_list
for i in range(1, len(stage_list)) :
	tmp_dict[stage_list[i]] = pattern_list[i]
num_cluster = 3
pattern_df = pd.DataFrame(tmp_dict)
pattern_df.set_index('PPCP', inplace = True, drop = True)
km = KModes(n_clusters=num_cluster, verbose=1,init='Huang', n_init = 4)
clusters = km.fit_predict(pattern_df)
pattern_df["cluster"] = clusters
pattern_df.to_csv("results_c2_cluster.csv", mode = "w", index = True)
data = pd.read_csv("results_c2_cluster.csv", index_col = 0)
data = pd.DataFrame(data['cluster'])
abraham_data = pd.read_csv(abraham_data_filename, index_col = 0)
tmp = scaler.fit_transform(abraham_data)
tmp = pd.DataFrame(tmp)
tmp.columns = abraham_data.columns
tmp.index = abraham_data.index
abraham_data = tmp
X = pd.merge(data, abraham_data, left_index = True, right_index = True)
y = X['cluster'].values
del X['cluster']
X.reset_index(inplace = True, drop = True)
kf = KFold(n_splits=5, shuffle = True, random_state = 42)
svm_acc_list, rf_acc_list, lr_acc_list = [], [], []
i = 1
for train_index, test_index in kf.split(X):
	X_train, X_test = X.loc[train_index], X.loc[test_index]
	y_train, y_test = y[train_index], y[test_index]
	svm_classifier = svm.SVC(kernel = 'rbf', probability=True, random_state=42)
	svm_classifier = svm_classifier.fit(X_train, y_train)
	svm_predicted_class = svm_classifier.predict(X_test)
	svm_acc_list.append(accuracy_score(y_test, svm_predicted_class))
	rf_classifier = RandomForestClassifier(random_state=42)
	rf_classifier = rf_classifier.fit(X_train, y_train)
	rf_predicted_class = rf_classifier.predict(X_test)
	tmp_acc = accuracy_score(y_test, rf_predicted_class)
	rf_acc_list.append(tmp_acc)
	lr_classifier = LogisticRegression(random_state=42)
	lr_classifier = lr_classifier.fit(X_train, y_train)
	lr_predicted_class = lr_classifier.predict(X_test)
	tmp_acc = accuracy_score(y_test, lr_predicted_class)
	lr_acc_list.append(tmp_acc)
	i = i + 1
acc_res = pd.DataFrame({'SVM' : svm_acc_list, 'RF' : rf_acc_list, 'LR' : lr_acc_list})
acc_res.to_csv("results_pred_acc.csv", mode = "w", index = True)
```
